# Development and Poverty Dynamics in Severe Mental Illness: A Modified Capability Approach in the Chinese Context

**DOI:** 10.3390/ijerph19042351

**Published:** 2022-02-18

**Authors:** Yue-Hui Yu, Man-Man Peng

**Affiliations:** 1 School of Public Administration and Policy, Renmin University of China, Beijing 100872, China; yuyuehui@ruc.edu.cn; 2 Institute of Advanced Studies in Humanities and Social Sciences, Beijing Normal University at Zhuhai, Zhuhai 519087, China

**Keywords:** development, severe mental illness, social protection, capability approach

## Abstract

Albeit poverty reduction has been listed as an overarching objective in many countries’ development plans, little is known about how development could shape poverty dynamics in disadvantaged groups. Guided by a modified capability framework, this study aimed to explore the long-term experiences of poverty dynamics in severe mental illness. Semi-structured interviews were carried out with 20 caregivers who provided care for persons with severe mental illness in Chengdu, China. Their perceptions on development, the illness, and social security were addressed. Content analysis was employed to analyze data. Participants experienced an overall improvement of life quality due to changes on urban infrastructure and transformed lifestyle. However, they were more disadvantaged while facing ability-based opportunities. These families were hindered from transferring opportunities into incomes. Negative impacts of the illness were also reflected in multiple stigma and conversion difficulties. Additionally, the high threshold for payment made those inclusive social security policies not inclusive for them. Poverty associated with severe mental illness was unlikely to be alleviated automatically within the process of development. Social isolation and high caregiving burden had aggravated poverty for those disadvantaged families. Poverty alleviation should be closely linked to the improvement in social policies in China.

## 1. Introduction

In recent decades, poverty reduction has been listed as an overarching objective in many countries’ development plans, especially those of developing countries [1]. There is also an increasing interest in understanding poverty dynamics shaped by development [2,3,4]. Studies show that development could be a double-edged sword on poverty reduction. On one hand, growth-led development has a trickle-down effect, which benefits poverty reduction through boosting income-earning opportunities and aggregating wealth for redistribution [5,6]. Development-oriented anti-poverty policies were highly favored and widely implemented around the world. Partly as a result, the international community has made remarkable achievements in reducing extreme poverty. On the other hand, new evidence has been acquired, which shows that development may not naturally lead to poverty reduction. Opportunities are less likely to be equally distributed throughout the whole population [7]. Some vulnerable groups have been structurally marginalized by the labor market, turning more disadvantaged within the development process. These groups include the elderly, the disabled, persons with severe mental illness, and many others [8,9]. Promoting development may be too simplified for a complicated issue like poverty, as it is still unclear about the form that development benefits might take, and the speed at which it might occur for disadvantaged groups [10].

Discussions related to the social effect of growth-led development in China resulted in a fundamental paradigm shift on poverty reduction. For a long time, development was taken as a core strategy to combat poverty in China. The government launched a whole package of programs to stimulate economic development after the reform and opening-up [11]. As a positive outcome of remarkable economic growth, China had contributed over 70% of the total global achievement on poverty reduction [12]. In recent years, more attention has been paid to disadvantaged groups that cannot ‘be developed’. Targeted poverty alleviation had dominated the anti-poverty discourse [13]. Guided by this new paradigm, appropriate anti-poverty responses should be varied according to the causes of poverty, the impoverished types, and specific development context. By the end of 2020, China had eradicated extreme poverty and stepped into a new era of alleviating relative poverty [14]. This targeted poverty alleviation paradigm is also applicable in the so-called post-poverty era. 

Whether, to what extent, and in what form disadvantaged groups can share the fruits of development constitute a series of fundamental questions for international poverty alleviation practices, as well as for China’s ‘common prosperity’ campaign [15]. In the era of targeted poverty alleviation, it is necessary to learn from those disadvantaged groups, to understand their living experiences and decision-making processes that may have kept them in disadvantaged positions or lifted them out of poverty [16,17]. However, there were few such studies, particularly in the Chinese context, where a distinct group of people remain relatively poor despite extraordinary social prosperity [18]. To achieve an in-depth understanding on how development may have benefited poverty alleviation for disadvantaged groups, this study applied qualitative research methods to explore the experience of poverty dynamics in severe mental illness (SMI). SMI is characterized as mental, behavioral, or emotional disorders that can result in serious disability [19]. There were over 16 million persons with SMI in China by 2009 [20]. People with SMI are at an increased risk of drifting into or remaining in poverty due to reduced productivity, increased medical costs, and loss of employment-associated earnings [21]. Because of strong stigma associated with mental illness, evidence shows that they are more disadvantaged than most other vulnerable groups [22,23,24]. 

Negative impacts of SMI may also occur at the household level, especially in societies that rely on families to provide patient care and financial support. Having a family member with SMI is associated with treatment costs, caregiving, and income loss [25]. In general, those households are less likely to benefit from the trickle-down effect of external development [8]. Accordingly, a wider range of people could be affected by SMI. Given the representativeness, a study to explore the experience of poverty dynamics in SMI can provide a good example to show how development in China may have shaped the fates of disadvantaged people, as well as how the government and society should further respond. In this study, the capability approach, proposed by Amartya Sen, was modified to fit the research objectives and context. Guided by this framework, research questions addressed in this paper included: (1) How did people live in SMI make sense of development and interpret the impacts of changes; (2) How did SMI exert negative impacts and keep people in long-term disadvantaged positions; (3) What was the role of social security on poverty alleviation for them? This paper first introduced the modified capability framework and then presented findings on each question. Two family cases were further elaborated to show the interactions of different forces in shaping poverty dynamics. Albeit in the same context, one family fell into poverty and another escaped.

## 2. Materials and Methods

### 2.1. Study Design

This study had a qualitative design with an inductive thematic approach. It was related to the Chengdu Mental Health Project (CMHP), a longitudinal project on mental illness and mental health services in Xinjin County, Chengdu [19]. Chengdu is the provincial city of Sichuan province. During the past few decades, Xinjin experienced huge development. Details of its achievements were described elsewhere [8]. Briefly, it had shifted from a middle-income rural county to one of the most favored investment places in Western China. Previously, a study using follow-up data demonstrated the poverty transitions for persons with SMI (*n* = 308) living in Xinjin for over 20 years (1994–2015). Overall, a larger proportion of them had fallen into poverty rather than escaped from it. Nevertheless, there were still 17.8% percent of them that managed to escape [21].

Based on what was known, this study was designed to further learn the experience of poverty dynamics behind the statistical numbers presented in the previous study. The capability approach proposed by Amartya Sen was applied and modified accordingly [26,27]. The term ‘capability’ refers to the array of social functions that a person, in a certain social context, could perform. One’s capability depends on the surrounding environment, the availability of resources (e.g., commodities from the market, public goods), and a set of personal characteristics (e.g., age, gender, and health status). Feasible utilization of these endowments forms the basic capability sets for individuals. Without the capabilities to convert potential endowments into commodities, one can be poor anytime [28].

**Figure 1:**
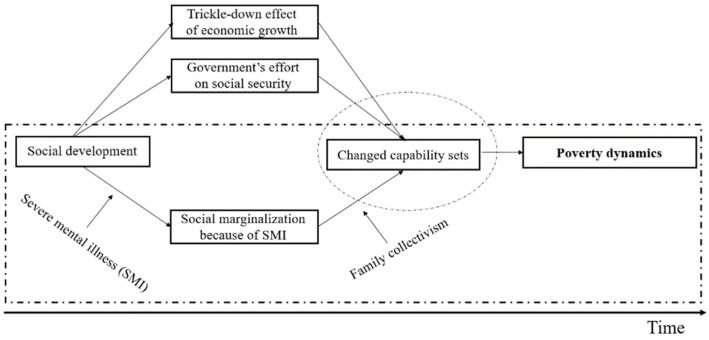
A modified capability approach to interpret poverty dynamics in SMI.

Figure 1 shows the modified framework for this study. Three major forces were considered for interpreting poverty dynamics in SMI. One was the trickle-down effect of fast economic growth, which had formed the surrounding environment [6]. The other was the government’s effort on social security, which may have improved the ‘availability of resources’ for persons with SMI and their families [29]. Social marginalization associated with SMI, as has been frequently mentioned [30,31], were viewed as the ‘group characteristics’, which had also played a role on shaping poverty dynamics.

### 2.2. Participants and Data Collection

**Figure 2:**
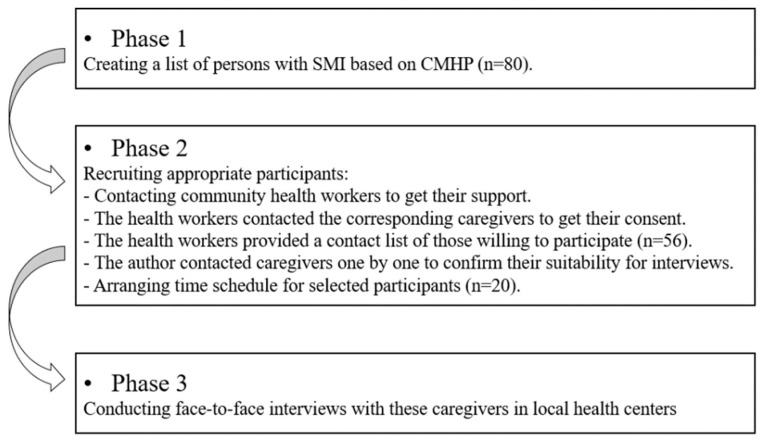
Procedures of the qualitative data collection.

Purposive sampling method was applied. Data collection was finished in three steps (Figure 2). First, a list of 80 out of 308 persons with SMI was created based on the CMHP dataset, with sufficient considerations on sampling diversity and confidentiality. Cases that never experienced poverty, experienced persistent poverty, escaped from poverty, and fell into poverty during 1994–2015 were all considered. Second, with the help from community health workers, the authors contacted the caregivers of listed persons with SMI. Caregivers were assumed to be more capable to share experiences on poverty dynamics, especially the negative impacts of caregiving. All interviews were arranged in local health-care centers. Given the distance, caregivers aged beyond 80 were excluded from the list of contacts. Some participants may fear losing face while being involved, and the confidentiality was considered. For each village, only one caregiver was recruited. Those who cannot communicate well were also excluded. Based on previous experience of conducting qualitative studies, normally, after finishing 15 interviews, little extra information can be obtained from a new one. As a conservative strategy, this study interviewed 20 caregivers, in order to make sure the research questions can be fully answered with sufficient confidence. Third, semi-structured interviews were held one by one with those caregivers. A consent form was read and explained before the interviews. Each interview took around one hour and was audio-recorded with a participant’s permission. A reflexive journal was kept throughout the study. 

### 2.3. Data Analysis

Content analysis was employed to analyze the data. A thematic approach was more pragmatism oriented, in which there were fewer rules to obey, thereby reducing the risk of confusion in matters of philosophical concepts and discussions [32]. Guided by the research framework, de-contextualization, re-contextualization, categorization, and compilation of the qualitative data were finished step by step [33]. Transcripts were coded using NVivo 12.0 software (QSR International, Doncaster, Australia), and assignment of codes was given repeated consideration.

### 2.4. Ethical Considerations

Ethical approval for this study was granted by the University Human Research Ethics Committee (EA1711060). Consent forms signed by participants were obtained. The form included a brief introduction of the research purpose, assurance of anonymity and confidentiality, the voluntary basis, and free options to withdraw. A separate sheet was used to record the participant’s name, age, contact number, address, and any other relevant information. This sheet was kept separately and only the researcher had access to it. The field work and data transcription were all finished by May 2019. Transcripts were anonymized and participants were ensured confidentiality. All research data were stored securely in concordance with the general rules of performing qualitative studies.

## 3. Results

Sample demographic characteristics of participants were presented in Table 1. They had been living in Xinjin County and had taken care of a person with SMI for over 20 years. Among them, twelve were male and eight were female. Their ages ranged from 42 to 78, with an average age of 58 years. More than half of them were spouses to persons with SMI (13 people), and there were also parents, adult children, and siblings. The average household size was three, measured by family members living and eating together. In terms of poverty typology, there were six households that fell into poverty, three escaped from it, six stayed in persistent poverty, and the remaining four remained non-poor. 

### 3.1. Perceived Development and Changes

Guided by the modified capability framework, this part reported caregiver’s perceptions on development, as well as its impacts. Their interpretations of development can be encoded into both changes on living environment and income-earning opportunities.

When asked about development there, most participants first thought of a transformed city landscape, including visible changes on buildings, roads, and factories, which formed a typical picture of urbanization. For the last decade or two, a large proportion of farmlands there were claimed for urban construction or contracted for large-scale cultivation. Many people moved into settlement apartments provided by the government. Rural lifestyle changed. People were more reliant on the market. Those changes enabled them to experience an overall improvement of life quality, as they felt happier, safer, and more convenient:

*Contracting land out not only reduced manual work, but also made me feel safe. Agricultural incomes can be greatly affected by the climate. Now, regardless of the climate, the income is more stable. That is the best part of becoming a city dweller. (No. 3).*

There were also some identity-based income sources that had contributed to improving their economic status. By saying identity-based, it means that anyone with a registered permanent residence (i.e., Hukou) in Xinjin is eligible to share the dividends of development policies. For example, as compensations of land acquisition, previous farmers were provided with resettlement housing and a monthly stipend. Many families were allocated more than one apartment, adding them extra income from leasing. For those who remained living in rural areas, the large-scale cultivation also increased their income. These benefits may be icing on the cake for others, but they were of paramount significance for households of a person with SMI. As participant No.16 noted:

*After our farmlands were claimed, every month I can receive around three thousand CNY. For a family like ours, this was the biggest revenue. Without it, I cannot image how hard our life could have been. (No. 16)*

Development in Xinjin also created more income-earning opportunities for local people. Compared with farming, these new opportunities required higher personal abilities. Because of functional impairment, persons with SMI were not fully capable of participating in the labor market. Their high demands on caregiving also hindered other family members from learning new skills and seizing those emerging opportunities. As a result, those trickled down opportunities had little positive impacts on them. Instead, it increased their income gaps with others.

*Those opportunities are for others, not for us. No matter how much others have earned, that’s their fates. I have a wife with schizophrenia. I must stay at home to provide care. Poverty is my fate. (No. 8)*

### 3.2. Long-Term Impacts of SMI on Poverty

In a society where improving economic conditions had created more opportunities for people to live better, households of persons with SMI remained disadvantaged. Apart from common recognized reasons such as income loss, medical costs, and caregiving burden, negative impacts of SMI were also reflected in multiple stigma and conversion difficulties for those families.

Caregivers experienced strong stigma and social isolation due to the disease. Worried about safety, their neighbors avoided interacting with those persons with SMI, and were also negative for other family members. They were even isolated from important kinship relations because their relatives felt ashamed. In the Chinese society, maintaining a social network was of paramount significance in reciprocating favors and earning income. SMI had resulted in a sharp reduction of social network, restricting their capabilities to access resources.

*I never bring friends home. This is a fatal blow to my business. Others cannot trust me without knowing my family. If they knew, I would also lose their trust. My brother is much richer than me. He can take his business partners home; they drink together and achieve consensus on cooperation in everyday interactions. I cannot. (No. 19)*

SMI and poverty were highly correlated. Having a person with SMI made families more likely to be poor within the development process. Poverty, in turn, became another source of stigma. Poor families were viewed as hopeless ones. They were discriminated by richer ones. Self-stigma on poverty further resulted in social withdrawal.

*We had richer relatives. I gave them gifts and was rejected. They despised me. They were afraid that I may ask them for help. I kept myself away from neighbors. Every time I visited them, I felt depressed to see them being better off. I had no social network. (No. 5)*

Families of persons with SMI also faced conversion difficulties. In case of having similar resources, they cannot achieve the same living standard as others. Instead, their weakness accumulated. For example, because of having a family member with SMI, a marriage-aged man with sufficient resources to start a normal family compromised to marry a disabled woman. It further increased the family dependency ratio and deteriorated the economic condition (No. 11). Another example was a school-aged man who was bullied by having a ‘freaky’ grandfather and dropped out. Without adequate education, he was not capable of getting a good job (No. 18). Overall, SMI ruined the sustainability of a family and made poverty pass down from one generation to the next. As Participant No. 7 noted:

*Mental illness may affect at least three generations. The illness on my mother affected her generation, mine and my kids’. When I was young, only my father worked to support the whole family. We lived in poverty and I dropped out from school early. As a result, I was not capable to find a well-paid job. I cannot work far away because I need to keep an eye on her. The current situation will inevitably affect my children. (No. 7)*

### 3.3. Complex Roles of Social Protection

Accumulated wealth enabled the government to strengthen its social security system. Those improvements can be categorized into targeted policies for persons with SMI and inclusive policies for general population.

In terms of targeted policies, guided by national policies on SMI management, persons with SMI were covered by a free medication scheme. Each year, they can take up to 800 CNY of free psychiatric medicine. After that, they need to pay for any extra fees. As an upgrade, the Sunshine Relief Project covered all outpatient and inpatient expenditures. However, its coverage was low. Although most participants showed their desperate needs, only two families had the patients being covered. Additionally, for those rated as a severe disability, there was also a supportive endowment insurance program and special subsidies like basic living allowance. If granted, their financial situation would be greatly improved. However, compared to their counterparts with physical disabilities, they were harder to be rated as a severe disability:

*My wife can hardly do any work. She was only rated as third-level disability and was not qualified for subsidies. I complained, the reason given was that she showed no obvious signs of disability. I think it’s not fair. My neighbor got a grant. He had his left hand amputated but he could do many things as usual. (No. 4)*

Compared with targeted policies, those inclusive programs like health insurance and endowment insurance were more beneficial, especially the endowment insurance. It was the main income source for many families of persons with SMI, and even the only income source for some of them. It had provided strong support for elderly life, and also partly relieved the younger generation. It should be noted that the endowment insurance did not shape poverty in a single direction. It had alleviated poverty for some families and aggregated it for others, at least in the short term. Benefiting from this scheme required payments in advance. Paying for insurance constituted a new poverty trap for some poor families, especially those where the family heads were in their 50s. They were less competitive in the labor market but pressured with insurance payment and other family duties. Instead of being protected, those families were even more vulnerable.

*I live worse than ten years ago. It gets harder to find a part-time job as I grow older. Meanwhile, the medical expenditure for both my wife and me are higher. My children are still young, sometimes I need to support them. I borrowed money to pay for the insurance. Now I am heavily in debt. (No. 8)*

### 3.4. Presenting the Full Picture: Two Case Illustration

In previous parts, three major forces shaping poverty dynamics in SMI (i.e., trickle-down effect of development, the social marginalization, and government’s effort on social security) were discussed separately. Here in this part, two retrospective stories were added to elaborate the interactions of these forces. Cases No. 1 and No. 19 were selected. Albeit in the same context, the former fell into poverty while the latter managed to escape. 

By the time of conducting this interview, Participant No. 1 was a 42-year-old unemployed woman. She lived together with her husband, daughter, mother (the person with SMI), father, and mother-in-law. Based on her narratives, their economic status improved from the early 1990s when there were more working opportunities. After their farmlands were contracted, both she and her husband worked in nearby factories. Their living conditions improved steadily until her father died in 2012. After that, she quit her job to provide full-time care for her mother. Their family income decreased. Meanwhile, their expenditures on elderly care, education, and endowment insurance payments increased. All those pressures drove the family into poverty. She faced strained relationships with others and experienced more physical and emotional problems.

**Figure 3:**
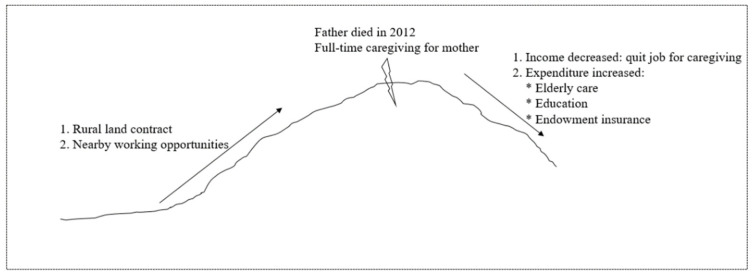
Process of falling into poverty for the No. 1 household.

Figure 3 summarizes key elements that had affected the poverty dynamics of the No. 1 household. This case reflected the vulnerability of households of persons with SMI. Although they may not be poor initially, a sudden change can drive them into poverty. In this family, the sudden change was the death of the participant’s father, which reshaped the family structure. With the increase of family burdens, the toll of SMI became apparent. 

**Figure 4:**
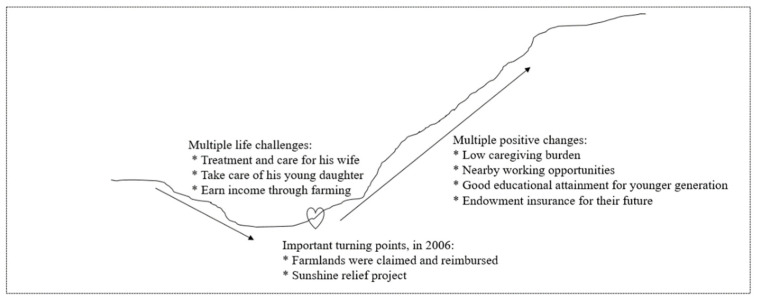
Process of escaping poverty for the No. 19 household.

In contrast, the No. 19 case showed how development and social security may help with pulling people out of poverty (Figure 4). Participant No. 19 was a 46-year-old man, living with his wife (the person with SMI), daughter, and son-in-law. In the beginning, he spent a lot on treatment for his wife, but it did not work. It drove the family into poverty. He had to shoulder multiple tasks on farming and caregiving. Life began to get better in 2006 when his farmlands were claimed for urban construction. As compensation, he was allocated an apartment and a one-off resettlement payment. His wife was also included in the Sunshine Relief Project and guaranteed her free treatment on mental illness. Once her illness got controlled, he was capable of working in nearby factories. Work income and the resettlement money enabled him to pay for endowment insurance and the daughter’s higher education. The daughter managed to have a decent job and a good marriage. Albeit still being affected by SMI, the family was less likely to be poor again.

## 4. Discussion

This study offered a meaningful framework to interpret the experiences and conceptualizations of development from the perspective of caregivers for persons with SMI in China. It modified the capability approach, a guiding framework in poverty studies, according to a specific context. The trickle-down effect of economic growth, negative impacts of SMI, and government’s effort to strengthen the social security system were examined from the participant’s perspective. An inductive thematic approach was applied to analyze their narratives. In general, development was beneficial for improving their life quality. However, caregiving burden, stigma, and poverty associated with SMI hindered their abilities to convert trickle-down opportunities into real income. Social security was expected to further alleviate poverty for disadvantaged groups. In reality, it played a complex role in shaping poverty dynamics. 

Although much work has discussed the relationship between development and poverty reduction [7,34,35,36], little is known about whether and in what form development is beneficial for pulling disadvantaged people out of poverty, as it can do for others [11]. Studies showed that the burden of SMI was very high for Chinese families during a period of fast development [37]. Albeit their mean income increased sharply within the development process, the wealth distribution had also become more unequal, thus the proportion of poor households increased instead [8]. As the long-term evidence on social drift accumulated [21], in what way the burden of SMI may have interacted with external development forces to shape poverty dynamics became a new question. This study applied qualitative research methods, unveiling the experiences of people who live through it.

Despite it being widely recognized that development can be beneficial for the poor [6], there were also numerous exceptions where it affected them negatively [1,7]. Drawing from experiences at the individual level, this study revealed that development did both good and harm to disadvantaged people. Development involved changes in infrastructure, lifestyle, and ways of earning income. The urbanization process had offered more identity-based income and improved the life quality for those persons with SMI and their family members. In this way, they were capable of sharing the dividends of development [38]. Nevertheless, a more developed society also required higher personal abilities, especially their work skills. The development context made employment an important arena in which SMI individuals and their families interacted [39]. SMI not only resulted in productivity and income loss for persons with SMI, but also their family members. They were called upon to provide care and thus were hindered from transferring income-earning opportunities into actual incomes. The bonus based on identities was far less than benefits from the market. Accordingly, although disadvantaged people can partly benefit from development, they lagged further behind.

Social security was expected to alleviate poverty through income redistribution [40]. In reality, its role in shaping poverty was complex. The effectiveness of social security in poverty alleviation had been doubted in many studies [41,42]. This qualitative study provided more details on how different types of social security programs may have increased the income gap instead of narrowing it for households of persons with SMI. Consistent with previous research, those inclusive social security schemes may not be inclusive as they ignored the existing disadvantages of certain groups [43]. For the endowment insurance program, people received returns based on their ability to pay. A relatively high payment threshold contributed to drive people into, or at least increased, their vulnerability of falling into poverty. People without payment ability were squeezed out from later returns and became poorer in their old age. Although special considerations were given for schemes that targeted persons with SMI, in general, they were far behind the demands.

In the Chinese context, a diagnosis of mental illness was a strong event for losing face, which could isolate a family from mutual help to maintain good life quality [44]. In addition to the stigma of mental illness, this study also highlighted the stigma of poverty. They mixed together, perpetuated, or at least amplified poverty in SMI in the long term. Guided by the modified capability approach, two cases were further elaborated in this study. Albeit in the same development context, case No. 1 fell into poverty while No. 19 escaped from it. Consistent with other studies [45,46], these two cases demonstrated the negative impacts of high caregiving burden on depriving the family from taking advantage of development opportunities. The illness did not necessarily lead to poverty. Instead, it increased the vulnerability of a household [43]. When it met other risk factors, such as excessive caregiving needs, accidents, and unemployment, those burdens may squeeze together and trap people deeply into poverty [47]. Chronic poverty is at the forefront of targeting poverty policy debates [2,48]. Compared to the past, current poverty reduction may be more difficult because those remaining in poverty are harder to reach. Further policy efforts to reduce poverty should not only focus on reducing the number of existing poor but also preventing the non-poor from becoming new poor in the later development process. The present study of poverty dynamics poses important implications for further targeted poverty alleviation practices. First, while it is still necessary to invest in creating opportunities for the poor, development policies should also focus on helping disadvantaged individuals to seize them. Policies on lowering caregiving burden associated with disadvantages could greatly enable family members to enjoy the trickle-down developmental opportunities [31]. Second, given that growth-oriented development was incapable of alleviating poverty associated with social marginalization, the role of social security should be further strengthened. Not only its coverage, but also the thresholds for disadvantaged groups should be reconsidered. Poverty alleviation should be closely linked to the development of social policy in China [31,46]. Third, findings on poverty dynamics suggest that the vulnerability of a household should be measured and poverty should be tackled in advance to reduce its intergenerational transmission. It calls for specific policy attentions on interventions to lower the stigma associated with SMI and poverty, allowing the family to build connections with others, develop support networks, and lift the next generation. 

Mental health issues have long been neglected in China’s health and development policies. While the burden associated with physical illness has been well documented [49], less is known about poverty associated with SMI [19]. This study, together with other studies rooted in the same context [8,21], revealed how SMI had deteriorated poverty for households of persons with SMI within a period of social prosperity. Although there was an increasing policy supply of social security for persons with SMI [50,51], the capability loss still largely hindered these households from the trickle-down effect of external development and kept them in chronic poverty [19,52]. As China has moved into the post-poverty era, formulating intervention plans and policies to target relative poverty associated with SMI may be crucial for achieving China’s new goals of inclusive development and common prosperity.

## 5. Strengths and Limitations

To the author’s knowledge, this was the first qualitative study that focused on poverty dynamics in SMI in the Chinese context. It was designed based on previous quantitative studies on the same study population [8,21], which may be better than most other independent-designed qualitative studies on its reliability. Nevertheless, there were still some limitations that should be mentioned. First, the research framework was modified based on strong highlights on economic growth, marginalization, and social security. Data collection and analysis were performed in an inductive thematic approach. Other potential significant forces in shaping poverty dynamics may have been neglected in this framework, for example, the development of social charity. Further studies may consider applying grounded theory to build a solid theoretical framework. Second, this study only recruited caregivers of persons with SMI as participants. The perceptions of social development and illness may vary from the patient’s perspective. Both persons with SMI and caregivers should be included in further studies, particularly paying attention to interpreting their different conceptions and opinions. Third, in the recruiting period, caregivers were selected based on their suitability for participating interviews. Therefore, the presented findings may be limited to unveiling the living situation of those being excluded.

## 6. Conclusions

This study was conducted in response to international calls arising to understand poverty dynamics in the development process and to identify ways of alleviating it, particularly for disadvantaged groups. It was the first attempt to unveil poverty dynamics in SMI, concerning a typical disadvantaged group, in China’s development context. In order to capture the complex nature of poverty dynamics, it modified the capability approach, and applied the qualitative method to examine the roles of development, illness, and social security in shaping poverty dynamics. Poverty associated with SMI was unlikely to be alleviated automatically within the process of development. Instead, social isolation and high caregiving burden may aggravate poverty for families of persons with SMI. The role of social security, a typical form of redistribution, was complex in the development context. Further thoughts on the question of ‘development for whom’ are strongly required.

## Figures and Tables

**Table 1:** Sociodemographic characteristics of participants.

	Caregiver Information N(%); M(SD)	Information of Persons with SMI N(%); M(SD)	Household Information N(%); M(SD)
Age (years)	58.15 (11.65)	58.85 (11.40)	
Gender, Female	8 (40%)	13 (65%)	
Employment			
Unemployed & retired	4 (20%)	13 (65%)	
Part-time work	9 (45%)	2 (10%)	
Farmer	7 (35%)	5 (25%)	
Marital status			
Have a spouse	18 (90%)	14 (70%)	
Have no spouse	2 (10%)	6 (30%)	
Relations to persons with SMI			
Parents	2 (10%)		
Spouse	13 (65%)		
Sons and daughter	3 (65%)		
Siblings	2 (10%)		
Household income per capita in 2018 (CNY)			28,620 (6676.36)
Household size in 2018			3.05 (1.10)
Poverty typology			
Persistent poverty			6 (30%)
Fell into poverty			6 (30%)
Escaped poverty			3 (15%)
Never experienced poverty			5 (25%)

## Data Availability

Interview guidelines and data are available on request from the corresponding author.
